# Electrochemical Method for the Assay of Organic Peroxides Directly in Acetonitrile

**DOI:** 10.3390/molecules30020374

**Published:** 2025-01-17

**Authors:** Vanina Ivanova, Mariya Pimpilova, Maria Stoyanova, Nina Dimcheva

**Affiliations:** 1 Department of Physical Chemistry, Plovdiv University “Paisii Hilendarski”, 24, Tzar Assen Str., 4000 Plovdiv, Bulgaria; vanina_1988@uni-plovdiv.bg (V.I.); marianas@uni-plovdiv.bg (M.S.); 2 Laboratory of Biologically Active Substances, Institute of Organic Chemistry with Centre of Phytochemistry, Bulgarian Academy of Sciences, 139 Ruski Blvd., 4000 Plovdiv, Bulgaria; mariya.pimpilova@orgchm.bas.bg

**Keywords:** composite catalyst, 2D nanomaterial, non-aqueous electroanalysis, organic hydroperoxides, peroxide value (PV)

## Abstract

Lipid peroxidation is a major process that determines the quality of various oil samples during their use and storage, in which the primary products are hydroperoxides (HP’_S_). HP’_S_ are very stable compounds at ambient conditions and are harmful to human health. Therefore, the evaluation of the degree of oil oxidation is an excellent tool for ensuring food safety. The peroxide value (PV) is the main parameter used for quality control in oils. Herein, we propose an alternative electrochemical method to the classical iodometric titration method most widely used for determining the PV. Our approach is based on the electrochemical quantification of hydroperoxides/peroxides in an organic solvent medium (acetonitrile and organic ammonium salt) using a composite electrocatalyst–glassy carbon electrode modified with 2D-nanomaterial graphitic carbon nitride doped with Co_3_O_4_. Calibration was made by the method of standard addition using benzoyl peroxide (BPO) as a model peroxide compound, dissolved in chloroform and added to fresh Rivana-branded anti-cellulite oil, used as a model oil sample. Calibration plots showed a linear response and the very good reproducibility of the analytical result (R^2^ ˃ 0.99). Further, in terms of accuracy, the method showed good results, since the BPO quantitative analysis was close to the theoretical response. In addition, the accuracy of the electrochemical method was compared with that of the standard iodometric titration method for determining the PV of vegetable fats (according to a standard method). Finally, using the electrochemical method, the concentration of peroxides was determined in a real sample—an anti-cellulite oil of the trademark Rivana with an expired shelf life.

## 1. Introduction

Organic peroxides are considered very harmful compounds because when they come in contact with bodily tissues, such as the tissue of the gastrointestinal tract, epidermis or mucous membranes, they tend to form free radicals responsible for the development of severe health problems such as chronic inflammation, ulcers or proliferating tumors. Therefore, the precise control of the peroxide content in commercial products containing lipids—pharmaceutical, cosmetic or food products—is of key importance [1]. The generation of organic peroxides ex vivo is due to the lipid peroxidation of oils, which are often also components of drugs, cosmetics or foods, as they are oxidized in the air in the presence of daylight [2]. This process of auto-oxidation is a free radical chain reaction and takes place in three steps, namely initiation, propagation and termination. The primary products of this process are hydroperoxides, which are relatively stable at room temperature and can be used to estimate the degree of lipid oxidation of oil samples [3]. The main indicator of primary oxidation processes in oils and of the amount of peroxides in oil-containing samples is the peroxide value (PV). The amount of peroxides indirectly serves to determine the rancidity of the oils due to their auto-oxidation [4].

There are different methods used for the quantitative determination of the PV, like instrumental and titrimetric approaches. Various rapid, selective and accurate instrumental techniques have been developed for estimating the PV in oil samples, such as spectroscopic, chromatographic and electrochemical methods [5]. The most widely used technique for the quantification of the PV is based on infrared radiation. Fourier transform infrared spectroscopy (FTIR) has attracted increasing interest in the last few years as it is non-destructive and requires minimal sample preparation [6]. FTIR is an excellent tool for the determination of peroxides in various oils, because the intensity of the bands in the infrared spectrum is directly proportional to the concentration of peroxides in the sample [7]. The powerful combination of FTIR and chemometric methods is often used for the estimation of olive oil freshness and oil oxidation, as well as for the monitoring of the fatty acid content in virgin olive oil [8,9,10]. The determination of the PV by FTIR uses different methodologies, such as the near-infrared spectroscopy region (4000–1200 cm^−1^) and FTIR with Attenuated Total Reflection (ATR) equipment [11]. The direct quantification of peroxides in oil samples by FTIR in NIR is difficult due to the low sensitivity and complex calibration pf the multivariate approach. However, FTIR with an ATR mode gives accurate results with a low signal-to-noise ratio, requires a small sample volume and is an excellent tool for viscous oil samples analyses [11]. However, the FTIR-ATR method may not be suitable for very viscous oils because a high viscosity can lead to difficulties in obtaining a reproducible sample thickness, which can affect the accuracy of the measurements [12]. In addition, one of the main disadvantages of the FTIR-ATR approach is its low sensitivity. This drawback can be overcome using multispectral identification methods, which are increasingly used in food analysis, e.g., the combination of FTIR and Raman spectroscopy [13]. FTIR spectra are generated by molecular vibrations and rotational frequencies, while Raman spectra are a result of molecular scattering, which is the inelastic scattering of incident IR light from the sample [14,15,16]. Thus, the multispectral FTIR–Raman identification technique could provide rich information about the content of oil samples, but the overlapping of analyte bands with those of other substances in the sample cannot be ignored. Therefore, it is necessary to combine FTIR and Raman spectroscopy with chemometric methods [17].

Among the instrumental techniques, UV-Vis spectroscopic approaches are widely used for determining the PV in edible oils. These approaches are frequently based on the oxidation of Fe (II) to Fe (III) by hydroperoxides in acidic medium, which then reacts with different reagents, resulting in color complexes such as Fe(III)-thiocyanate and Fe(III)-xylenol orange, absorbing light in the visible region of the electromagnetic spectrum [18]. These methods are simple and low cost, but their accuracy is easily affected by the content of carotenoids in oils. Hence, it is necessary to extract carotenoids from the solution containing Fe(III)-thiocyanate or Fe(III)-xylenol orange complexes using organic solvents before PV determination [17,19].

Fluorescence and chemiluminescence are emerging spectroscopic techniques used for the qualitative and quantitative determination of organic peroxides in oil samples due to their high sensitivity, rapidness and robust information content [5]. In addition, fluorescence spectroscopy (FS) has significant advantages in terms of its wide dynamic range, selective detection and non-destructiveness. However, several shortcomings of FS limit its practical applications, such as specific sample preparation through extraction or derivatization and the need for specific excitation sources [16]. In contrast, in the chemiluminescence method, no external light source for excitation is used, which simplifies the instrumentation. Additionally, sample preparation using chemiluminescence spectroscopy (CS) is not so complex, and this technique allows the real-time monitoring of changes in peroxide levels during reactions. The main drawback of CS is the need to carry out specific chemical reactions that produce light, which also limits the application of the method to different oil samples.

More recent research has focused on applying ^1^H nuclear magnetic resonance (NMR) spectroscopy for PV determination in edible oils, because of its simplicity and time efficiency [5]. The chemical specificity of the NMR spectrum is the major advantage of this technique over other spectroscopic methods [16], as the NMR (^1^H) spectra of fatty acids and their derivatives can be fully assigned [14,15,20]. However, all currently available instruments based on the NMR technique use expensive high-field super-conducting magnets, as well as probes requiring cryogenic cooling and specially trained personnel to operate the instrument. Hence, none of the abovementioned spectroscopy-based instrumental methods seem to be suitable for routine and low-cost analysis in industrial settings [16].

Among the methods used to determine the PV of oil samples, chromatographic techniques such as gas (GC) and high-pressure liquid chromatography (HPLC) are characterized by a number of advantages, including excellent selectivity, a low LOD, a wide detection range and low sample consumption [21]. However, some major shortcomings limit the practical application of chromatographic techniques for real-time oil oxidation monitoring, such as the high cost of instruments, complex and time-consuming sample preparation before GC and HPLC, the high cost of analyzing a single sample, as well as the duration of the work and the complexity of data processing after HPLC [22].

All considered instrumental techniques are accurate, rapid and sensitive, but require relatively expensive instrumentation and highly trained personnel.

The method most frequently used in laboratory practice in Bulgaria for PV determination is the conventional method of iodometric titration in a mixed aqueous-non-aqueous medium. This approach is widely applied as a classical analytical method due to its simplified operation and low hardware requirements [23]. The method is based on the reaction between peroxides in a sample with KI, resulting in the formation of molecular iodine, which is titrated using a sodium thiosulfate solution. Starch is used as an indicator, which changes the color of the solution from colorless to blue–violet in the presence of iodine, which is released from the sample. When titrating the water sample with sodium thiosulphate, the sample is discolored when the equivalence point is reached (BDS EN ISO 3960-2017) [24]. Although the titrimetric method is simple, low-cost and routine, several shortcomings restrict its application, such as the need for special sample preparation (dissolving oil samples in organic toxic solvents—trichloromethane, toluene and ether, extraction, and titration in mixed medium), and the nature of the reagents, the temperature, extraction time, etc. In the titration of crude oils, the equivalence point is difficult to distinguish because of the dark color of the oil samples [23,25]. Other disadvantages are the high LODs and narrow detection range [23].

In recent years, electrochemical techniques belonging to the group of instrumental approaches have attracted increasing interest due to their simplicity, low-cost, high sensitivity, selectivity and portability [26,27,28,29,30]. A relatively new electrochemical method for PV determination in edible oils is based on a change in electrical conductivity in an aqueous medium during a reaction between KI and hydroperoxides in the oil sample [6]. This approach has high reproducibility, but its accuracy is low. To perform the in situ monitoring of lipid oxidation and the miniaturization of the instrumentation, electrochemical portable sensors like electronic nose (E-nose) and electronic tongue (E-tongue) have been developed. The latter represent simple, rapid, inexpensive and highly efficient portable miniaturized devices for lipid oxidation monitoring [31]. The E-nose method was used to study the flavor qualities of oils, while the E-tongue approach was applied to monitor the PV of olive oil during storage [32,33,34]. However, such portable miniature devices are still in the early stages of research. Further research is needed on their long-term storage stability and biocompatibility [23].

In recent decades, electrode modification techniques have been intensively developed for various applications, such as solar energy conversion and storage [35], selective electro-organic synthesis [36], molecular electronics [37], electrochromic display devices [38], protection from corrosion [39] and electroanalysis [40]. The rapid development of experimental chemistry and the desire of scientists to develop fast and accurate methods for determining the extent of oil oxidation have led to the idea of introducing emerging materials such as 2D disulfides of transition metals, metal–organic frameworks, covalent organic frameworks, halide perovskites, etc., into the basis of various analytical techniques, including the modification of electrode surfaces [41].

After the discovery of monolayer graphene in 2004 [42], 2D carbon-based nanomaterials have attracted the attention of many researchers in various fields, ranging from electronics to biomedicine [43]. Their unique catalytic properties and high surface-to-volume ratio make them suitable for a wide range of applications, such as environmental catalysis [44], drug delivery carriers [45], sensing and energy applications [46]. Often, carbon-based nanomaterials are combined with other materials, resulting in nanocomposites that significantly improve the sensitivity and selectivity of modified surfaces because of the synergistic effect between them. These 2D nanomaterials provide features such as an increased electroactive surface area, additional catalytic centers, and a tunable structure [47]. It was found that the addition of nitrogen atoms is the most effective approach to improving the electrocatalytic activity of carbon materials [48]. Carbon-nitride-based compounds with a high N-to-C ratio are new-generation materials that are used to develop electrocatalysts [49]. Graphitic carbon nitride (g-C_3_N_4_) is a non-metallic, graphene-like 2D material composed of covalent bonds (C-N), forming a π-conjugated polymer with a high molecular weight and unique electronic structure that determine its semiconductor properties [50]. g-C_3_N_4_ is a multifunctional, heterogeneous, non-metallic catalyst [51], the main drawback of which is its low specific surface area. A known approach to increasing the specific surface area of g-C_3_N_4_ is its doping with transition metal oxides in the form of ultrafine dispersion, which distribute between g-C_3_N_4_ nanosheets and prevent their agglomeration [52,53]. Transition metal oxides (TMOs) possess good electrical and photocatalytic properties due to their size, shape and larger surface area [52].

An earlier study by our group demonstrated that a composite comprising Co_3_O_4_-g-C_3_N_4_ and Nafion used as a modifying phase of glassy carbon surfaces possesses excellent electrocatalytic activity during the reduction of water-soluble hydroperoxides [54]. Later work dealt with the examination of the electrocatalytic activity of pristine or TMO-doped carbon nitrides, while previous work explored both the characterization of Co_3_O_4_-doped g-C_3_N_4_ by TEM, XRD, FT-IR, ICP-OES and BET, and its heterogeneous catalytic activity in peroxide bond disruption [55]. A recent review summarizing advances in the use of electrode materials in hydrogen peroxide sensing [56] certified that carbon-based materials, such as graphene or reduced graphene oxide, carbon nanotubes and mesoporous carbon, are the preferred materials for H_2_O_2_ sensing and biosensing. However, nanoparticles of noble metals (Pt, Pd, Au, Ag), metal oxides, and polymeric materials, e.g., conducting or dendritic polymers, molecularly imprinted polymers or biomacromolecules, have also been applied to this end.

Unlike hydrogen peroxide, most naturally formed hydroperoxides are insoluble in water and their direct evaluation must be performed under non-aqueous conditions. Therefore, the aim of this study is to explore the electrochemical activity of composite-modified glassy carbon electrode in acetonitrile with respect to water-insoluble peroxides and hydroperoxides, and to develop a simple method with rapid response for the quantification of organic peroxides directly in a non-aqueous medium. For the characterization of the analytical performance of the discussed electrocatalytic method, classical iodometric titration was used as a reference.

## 2. Results

### 2.1. Optimisation of the Operating Conditions

Electrochemical studies in a non-aqueous medium have several limitations, including low conductivity, the limited solubility of electrolyte salts, high noise, and the impossibility of using conventional reference electrodes; however, non-aqueous electrochemistry allows the redox properties of some water-insoluble organic compounds to be studied. Keeping this in mind, further electrochemical studies were performed in the aprotic organic solvent acetonitrile containing 0.1 M of the electrochemically inert electrolyte tetrabutyl ammonium perchlorate (TBAP) to increase the electrical conductivity of the medium.

**Figure 1:**
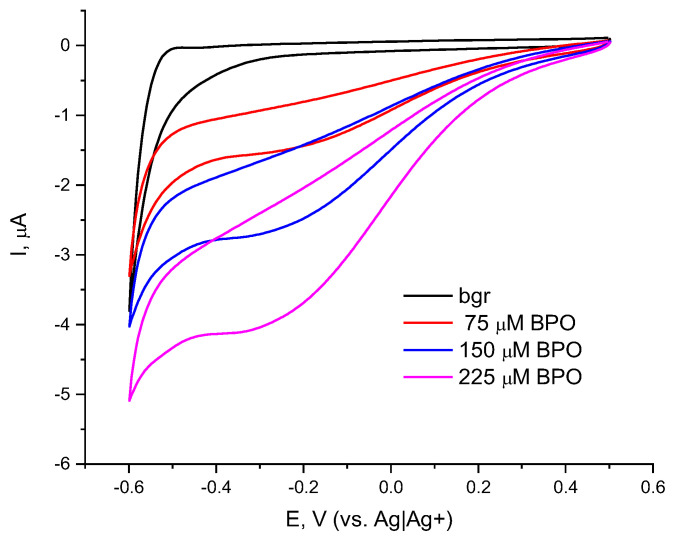
Cyclic voltammograms of Co-g-C_3_N_4_/Nafion/GCE in the absence and presence of BPO/ACN; background electrolyte 0.1 M TBAP in acetonitrile and reference electrode Ag|Ag^+^; scan rate of 20 mV/s.

The electrochemical behavior of the Co_3_O_4_-doped g-C_3_N_4_/Nafion-modified electrode (Co-g-C_3_N_4_/Naf) was investigated by cyclic voltammetry. Figure 1 compares the cyclic voltammograms (CVs) of the electrode–catalyst recorded in the absence and presence of benzoyl peroxide (BPO). This peroxide was chosen as a model analyte because it is a non-hygroscopic solid and its solution in organic solvents can be prepared with exact concentrations; it can hence be used as an analytical standard. The background CV, i.e., the voltammogram recorded in the absence of peroxides (black solid line), suggests that the catalytic layer possesses catalytic activity towards oxygen, which is dissolved in the acetonitrile, as the I-E curve possesses an “oxygen tail” appearing over the potential region between −0.4 and −0.6 V vs. Ag|Ag^+^.

When portions of BPO standard solution are added to the electrolyte in the cell, an electrochemical reduction of the peroxide occurs (red, blue and purple solid lines). When scanning the BPO present in the negative direction, a reduction wave is observed, starting at a potential of 0.3 V (vs. Ag|Ag^+^ pseudo-reference electrode) and reaching a broad peak at −0.2 V, which, upon further changes in the potential, passes into a second wave of reduction. As the concentration of the model peroxide increases, the reduction maximum gradually shifts to more negative potentials.

As suggested by Vasudevan [57], the reduction of peroxides and hydroperoxides in aprotic medium (i.e., lacking mobile protons) proceeds via a mechanism substantially different from the reduction of H_2_O_2_ in aqueous medium, which can be schematically represented as follows:

R_1_-O-O-R_2_ → R_1_-O^−^ + R_2_-O^−^ (1)

R-O-O-H → R-O^−^ + OH^−^ (2)

Furthermore, slow protonation of the formed anions from the supporting electrolyte may take place until the corresponding alcohols form.

**Figure 2:**
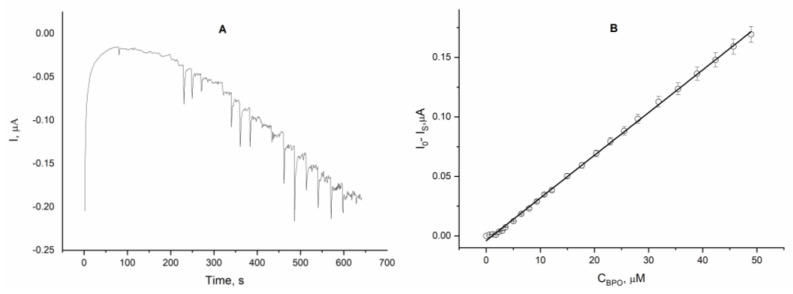
Chronoamperometric record (**A**) and calibration plot (**B**) of an electrode modified with Co-g-C_3_N_4_/Naf when aliquots of 0.003M BPO were added; background electrolyte: 0.1 M solution of TBAP in acetonitrile and reference electrode Ag|Ag^+^; working potential of −0.2 V.

The polarization dependencies suggest the possibility of quantifying the selected organic peroxide by means of chronoamperometry at a constant working potential of −0.2 V, which can be achieved based on a calibration plot. Chronoamperometric experiments performed at a constant potential of −0.2 V (vs. Ag|Ag^+^) showed a stepwise change in the reduction current when portions of peroxide were added, reaching steady-state values within 10 s (Figure 2A). The calibration graph obtained based on the chronoamperometric record shows the linear dependence of the signal versus the peroxide concentration up to 50 μM. It should be noted that at low concentrations (up to about 2 µM), a deviation from linearity is observed, which is probably due to the weak electrode signal over this concentration range, which in turn leads to considerable errors in the determination.

The calibration equation obtained from the linear regression analysis is as follows:

I_S_ − I_0_ = (−3.96 × 10^−9^ ± 4.95 × 10^−10^) + (3.59 × 10^−3^ ± 2.13 × 10^−5^) × C_BPO_

where I_S_—steady state current recorded in the presence of peroxide, µA;

I_0_—background current, µA;

C_BPO_—concentration of BPO, µM.

**Figure 3:**
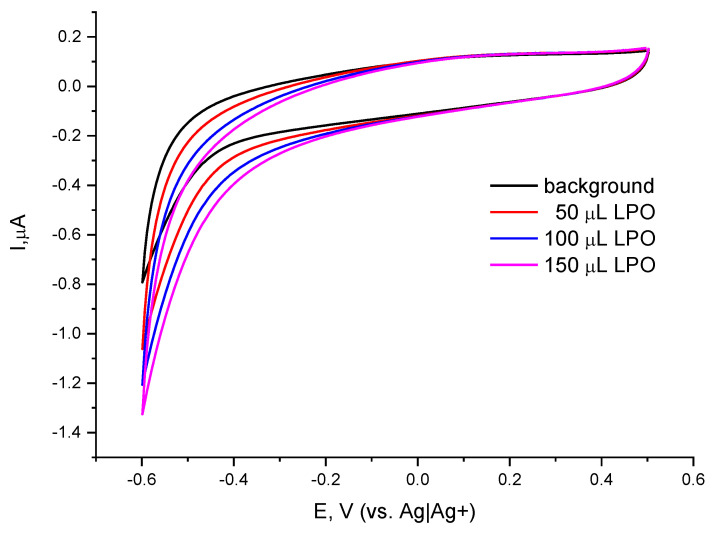
Cyclic voltammograms of Co-g-C_3_N_4_/Nafion/GCE in the absence and presence of lauroyl peroxide (LPO) in ACN; background electrolyte 0.1 M TBAP in acetonitrile and reference electrode Ag|Ag^+^; scan rate of 20 mV/s.

Keeping in mind that the designed peroxide electrode was to be used for assaying the peroxide value (PV) in vegetable oils, several other peroxides were tested as possible standards for the quantification of peroxides under non-aqueous medium by applying the same approach. Figure 3 depicts the cyclic voltammograms of the catalytic peroxide electrode in the absence and presence of varying concentrations of dilauroyl peroxide, a solid aliphatic peroxide compound. The CVs recorded show a similar behavior to that observed under equivalent conditions in the presence of BPO as a model peroxide. However, the electrochemical reduction of the aliphatic peroxide compound took place at a much lower rate at the chosen operating potential of −0.2 V, which indicates that the reduction proceeds with a considerable overpotential compared with the electrochemical reduction of benzoyl peroxide. It is plausible that the bulkier molecule of dilauroyl peroxide adsorbs more on the electrode surface, thus largely blocking it. These results indicate that the choice of BPO as a standard for calibration is reasonable, so further studies were implemented using BPO standard solutions.

**Figure 4:**
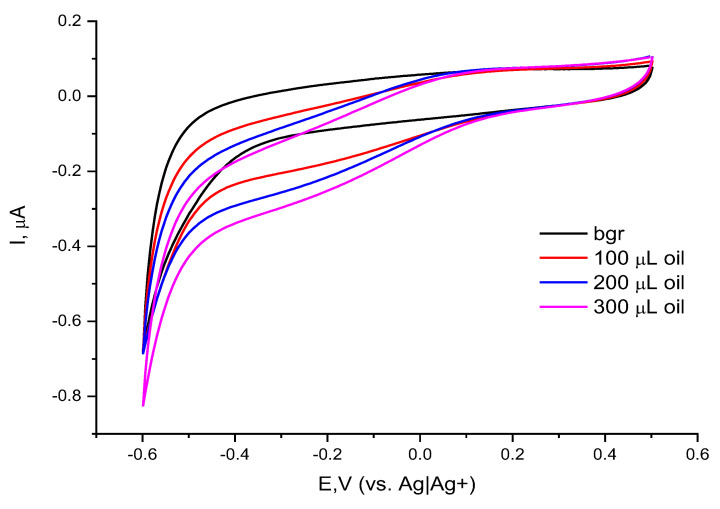
Cyclic voltammograms of Co-g-C_3_N_4_/Nafion/GCE in the absence and presence of oil (dissolved in CHCl_3_); background electrolyte 0.1 M TBAP in ACN and reference electrode Ag|Ag^+^; scan rate of 20 mV.s^−1^.

Furthermore, the catalytic peroxide electrode was used to determine the concentration of peroxides in a real sample, using a mixture of expired vegetable and essential oils (anti-cellulite massage oil—Rivana, Bulgaria). For this purpose, the electrochemical behavior of the electrode–catalyst upon the addition of anti-cellulite oil dissolved in chloroform (CHCl_3_) was investigated by cyclic voltammetry over a range of potentials from −0.6 to 0.6 V (vs. Ag|Ag^+^). The addition of aliquots of the real sample solution to the working medium resulted in a pronounced reduction wave (visible in the reverse scan) starting at a potential of 0.18 V and reaching a plateau between −0.2 V and −0.4 V (Figure 4). The presence of this reduction wave gave us a reason to further explore the opportunity to determine the amount of organic peroxides in the real sample by means of chronoamperometry at a constant potential.

**Figure 5:**
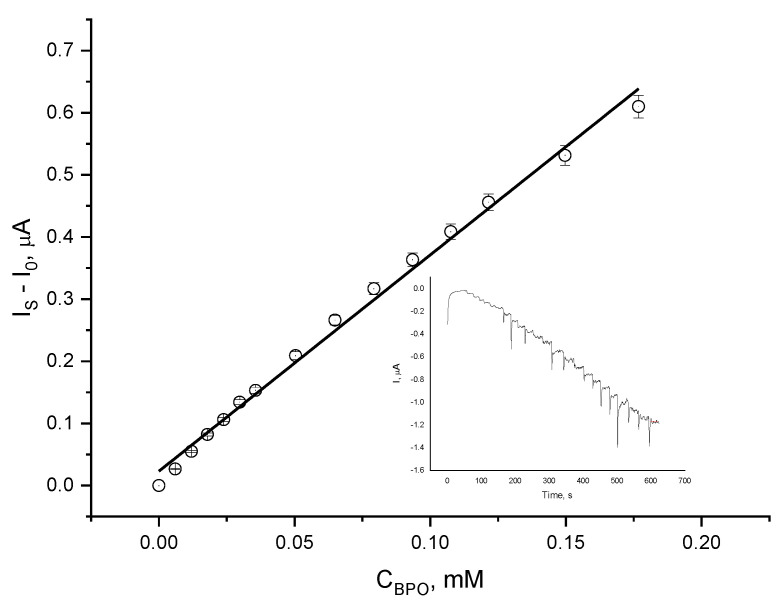
Chronoamperometric record (inset) and calibration plot of a Co-g-C_3_N_4_/Nafion-modified electrode when aliquots of 0.003 M BPO were added (dissolved in CHCl_3_/oil); background electrolyte: 0.1 M TBAP in ACN and reference electrode Ag| Ag^+^; potential of −0.2 V.

Chronoamperometric measurements were performed (Figure 5, inset) at a constant potential of −0.2V (vs. Ag|Ag^+^) while adding aliquots of the standard peroxide (BPO) solution to the operating medium. The calibration plot shows the linear dependence of the electrode response versus the BPO concentration (correlation coefficient of 0.993). To account for the effect of the real sample constituents, the standard 3 mM of BPO solution was dissolved in chloroform, to which 0.4 g of fresh non-peroxidized oil was added.

### 2.2. Analytical Performance of the Catalytic Peroxide Electrode

**Figure 6:**
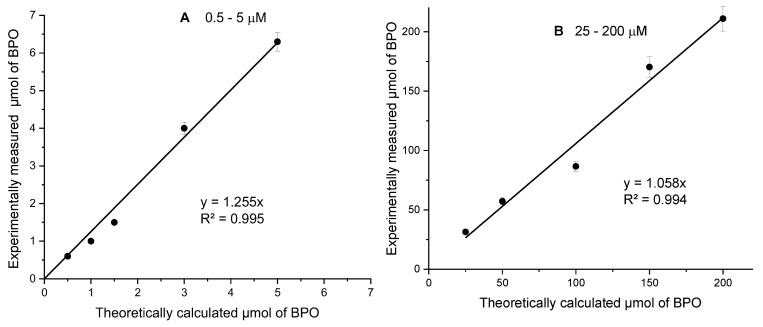
Correlation dependencies of measured versus theoretical amounts of peroxide (BPO) in a real oil sample enriched with peroxides in a narrow (0.5–5 μM) (**A**) and in a wider (25–200 μM) (**B**) range of peroxide concentrations (in μmol L^−1^). The regression equations and the regression coefficients R^2^ are provided in the lower right part of the Figures.

To evaluate the accuracy of the electrochemical method for the determination of the PV, calibration standard solutions of BPO as a model peroxide were prepared using low (1, 2, 3, 6, 10 μM) and high (50, 100, 200, 300, 400 μM) peroxide concentrations. The signals of all standard solutions of BPO in CHCl_3_ were measured. For this purpose, 0.5 mL was taken from each standard solution and nearly 0.4 g of fresh anti-cellulite oil was dissolved in them. The results obtained (Figure 6) show good agreement between the experimentally obtained values and the theoretically calculated ones, with high correlation coefficients (R^2^ = 0.995 and R^2^ = 0.994, respectively).

The slopes of the linear dependencies (1.255 and 1.059, respectively) indicate the deviation of the measured values from the expected ones, with 25.5% for the low concentrations (from 0.5 to 5 μM) and 5.9% for the range from 25 to 200 μM. These results indicate that if the oil contains 2.0 mmol/kg peroxides, 2.51 mmol/kg will be detected in the low concentration range (˂5 µM). Similarly, if the oil contains 20 mmol/kg peroxides, 21.2 mmol/kg will be detected in the concentration range of 25–200 µM. On the other hand, the response is linear and shows the good reproducibility (R^2^ > 0.99) of the analytical result.

**Figure 7:**
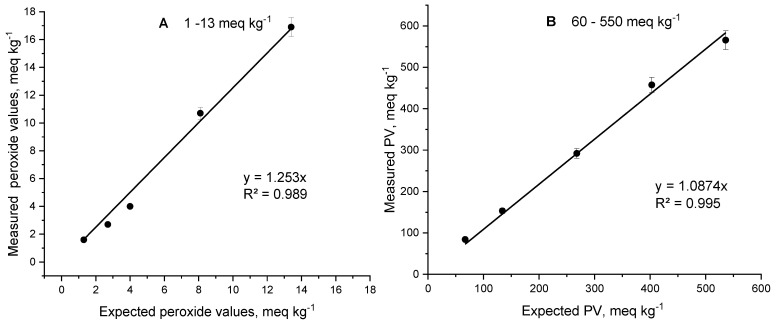
Correlation dependencies of measured versus expected amounts of O_2_ in a real oil sample enriched with benzoyl peroxide in a low (1–13 meq kg^−1^) (**A**) and high (60–550 meq kg^−1^) (**B**) range of peroxide concentrations, expressed in meq/kg. The regression equations and the regression coefficients R^2^ are provided in the lower right part of the Figures.

Further, the expected and measured μmol BPO levels were converted into expected and measured peroxide values (PVs), respectively, which are compared in Figure 7.

A similar positive deviation between the measured and expected values for the PV was observed, with the PV being 25.3% for the low concentration interval and 8.7% for the wide.

**Figure 8:**
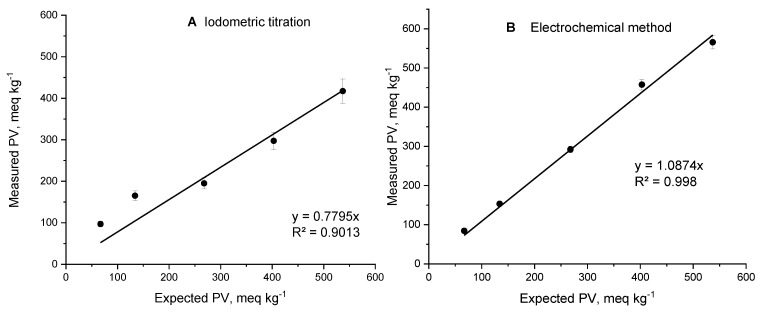
Measured vs. expected peroxide value of a real sample spiked with known amounts of BPO analyzed using the titrimetric method (BSS EN ISO 3960:2017) [24] (**A**) and using the electrochemical method (**B**).

The accuracy of the electrochemical method in a wide concentration range was compared with that of the conventional titrimetric approach for the determination of the PV. For this purpose, known amounts of the BPO spiked at different concentrations in fresh anti-cellulite oil were measured. Regardless of the concentration of BPO, the electrochemical response was linear and showed good reproducibility (R^2^ > 0.99). In contrast, the iodometric titration for all BPO amounts demonstrated significant deviations between the measured and expected peroxide values (0.7795 was the line’s slope in the iodometric titration, in comparison with 1.0874 in the electrochemical method) (Figure 8) (Table 1). These results imply that if the oil sample contains 20 mmol/kg peroxides, 15.6 mmol/kg peroxides will be detected by iodometric titration and 21.7 mmol/kg peroxides will be detected by the electrochemical method. It is likely that the low reproducibility of the titrimetric method compared to the electrochemical one is due to the extraction step of the sample pretreatment, which is part of the titrimetric method.

**Figure 9:**
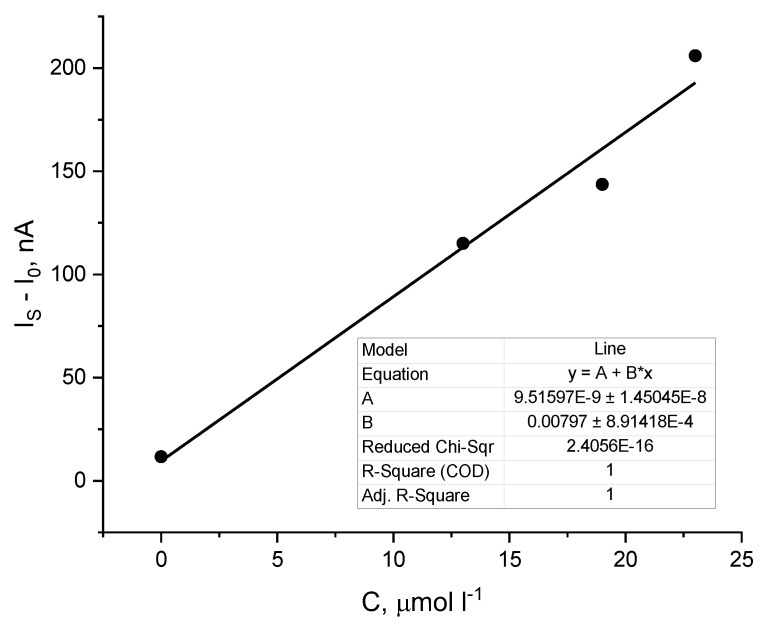
Determination of the PV of the real sample by the method of standard addition: after adding the sample, three more aliquots of the external standard solution were added (standard peroxide solution, BPO, in chloroform, to which the real sample was added) to the electrochemical cell. Below are provided the regression model and the corresponding regression coefficients calculated by least squares method.

Finally, the peroxide value of the real sample containing a mixture of easily oxidizable vegetable oils was determined by the standard addition method. According to this method, the intercept of the calibration line corresponds to the signal of the real sample (Figure 9). Dividing this signal by the slope of the line, one obtains the concentration of peroxides in the electrochemical cell of 1.25 × 10^−6^ M. Considering the dilution and mass of the real sample, we find that the real sample contains 276 μmol of peroxides, which corresponds to PV = 739.75 ± 64.36 meq O_2_ kg^−1^. Therefore, the vegetable oils contained in the real sample are highly oxidized.

## 3. Discussion

Iodometric titration is an easy way to determine the PV. However, this method is labor-intensive, time-consuming, and requires a lot of reagents and large amounts of sample and organic solvents. In addition, its accuracy and reproducibility strongly depend on the reaction conditions, e.g., light, temperature, the skills of the operator, the sodium thiosulfate decomposition, etc. Furthermore, the iodine released in the organic phase can participate in side reactions, such as oxidation, by being dissolved in the solution oxygen or by interacting with unsaturated lipids. These interfering processes can lead to an inadequate estimation of the PV.

Unlike iodometric titration, the electrochemical method developed by our research group allows the analysis of lipid samples directly in acetonitrile. Its accuracy and reproducibility far exceed those of iodometric titration, and omitting the extraction step considerably shortens the length of sample preparation. Due to the simplicity of operating with electrical signals and the compact size of the equipment, the electrochemical method represents a good alternative to traditional instrumental analysis (e.g., by HPLC or FTIR), providing the simple, accurate, rapid, and reproducible analysis of the PV with very small amounts of sample required. In addition, our electrochemical approach ensures high accuracy over a wide range of PVs (from 1 to 540 meq kg^−1^). Hence, the analytical method developed based on the catalytic peroxide electrode has significant potential to be used as an alternative to most of the known methods for determining the peroxide values of lipid-containing samples.

## 4. Materials and Methods

### 4.1. Materials

The following reagents and chemicals were used in the present study: dibenzoyl peroxide, p.a., dilauroyl peroxide, p.a., dicumyl peroxide, p.a. and di-tertbutylperoxide, p.a., (Acros Organics) used in the form of solutions with an initial concentration of 0.3 M or 3 mM in chloroform; Nafion™ 117, as a 5% suspension in a water–alcohol mixture (Sigma-Aldrich, Saint Louis, MI, USA); the ultrapure solvents acetonitrile (CH_3_CN) and chloroform (CHCl_3_), with specification p.a. (Fisher Chemical, ThermoFisher Scientific, Waltham, MA, USA); and quaternary ammonium salt (tetrabutylammonium perchlorate—TBAP, Acros Organics, Carlsbad, CA, USA).

Anti-cellulite oil from the Rivana brand (Bulgaria) was tested as a real sample. Both fresh and expired products were used. The mixture contained the following: *Vitis vinifera* Seed Oil, *Citrus paradisi* Peel oil, *Cimbopogon schoenanthus* Oil, *Rosmarinus officinalis* Oil, *Juniperus communis* Oil, Linalool, Limonene, Geraniol and Citral.

Electrode material: Commercially available glassy carbon materials were used as working electrodes. The electrodes were cylinders of chemically resistant polymers with a length of 6 cm and an outer diameter of 6 mm, in which there was an embedded cylinder of glassy carbon with a diameter of 3 mm (visible surface area 0.071 cm^2^, manufactured by CHI, Austin, TX, USA) or with a diameter of 2 mm (visible surface area 0.031 cm^2^, manufactured by Metrohm, Utrecht, The Netherlands). The glassy carbon material was connected to a metal current lead, which connected it to the electrochemical apparatus.

### 4.2. Apparatus and Measurments

All electrochemical measurements were performed in a conventional three-electrode cell (working volume 20–100 mL, Metrohm-Autolab, Utrecht, The Netherlands), with a catalyst-modified disc of glassy carbon (GCE) used as the working electrode, Ag|Ag^+^ used as the reference electrode and a platinum foil used as the auxiliary electrode. the electrochemical workstation PGSTAT Autolab 302N (Metrohm, Utrecht, The Netherlands) with Nova 2.1.6 software was used in all experiments. Cyclic voltammograms (CVs) were recorded at a scan rate of 20 mV s^−1^. Chronoamperometric measurements were performed under constant stirring.

### 4.3. Synthesis of Co-Doped g-C_3_N_4_

The synthesis of the Co-doped g-C_3_N_4_ was carried out via thermal polycondensation on protonated melamine in the presence of cobalt salts, which were further thermally decomposed to a spinel cobalt oxide (Co_3_O_4_) [58]. To reach 5% of Co by weight in the final catalyst, a certain amount of Co (NO_3_)_2_·6H_2_O was dissolved in 50 cm^3^ of methanol and added to the protonated melamine, under continuous stirring at 50 degrees. until the evaporation of the MeOH. The obtained solid was dried at 60 °C for 10 h and calcined at 550 °C for 2 h.

### 4.4. Modification of Working Electrode

The modifying phase was prepared as described previously [54]; in brief, 1–2 mg of graphitic carbon nitride, doped with tricobalt tetroxide (with a Co_3_O_4_ content of 5 wt.% to 8 wt.%), was added to 1 mL of an aqueous suspension of the ionomer Nafion^TM^ (with a concentration of 0.2%) and dispersed ultrasonically for 30 min. Then, a 5 µL drop of the resulting composite electrocatalyst was applied to the surface of the cleaned glassy carbon electrode and air-dried for 16–24 h at room temperature.

The Co-g-C_3_N_4_/Nafion catalytic electrode can be used to perform analysis up to 10 times within 1 day and stored for one year or more. It is stored dried, after being washed with ultrapure water, in the air and at room temperature, and is capped with a plastic protector. Before use after long storage (more than 3 days), the catalytic electrode is reactivated by placing it in an electrolysis cell filled with background electrolyte (0.1 M tetrabutylammonium perchlorate dissolved in acetonitrile), connecting it in a three-electrode configuration, together with reference and auxiliary electrodes, to the electrochemical workstation and treating it by cyclic voltammetry over a potential ranging from −0.4 V to +0.6V for at least three cycles, after which it is calibrated twice according to the description (Section 4.6). The background electrolyte with a concentration of 0.1 M was prepared by dissolving tetrabutylammonium perchlorate in acetonitrile.

### 4.5. Study of the Electrocatalytic Activity of Co-g-C_3_N_4_/Nafion—Modified GCE

The Co-g-C_3_N_4_/Nafion-modified GCE working electrode, the reference electrode Ag|Ag+, and the platinum foil auxiliary electrode were positioned in a standard single-compartment electrochemical cell with a working volume of 10–50 mL. 10 mL of the background electrolyte 0.1 M tetrabutylammonium perchlorate, dissolved in acetonitrile, is added and the electrodes are connected to a DC source. The set DC voltage is controlled through the electrochemical workstation and the current strength is measured. The working potential is set to −200 mV and the establishment of a stationary background current of about 0.2–0.5 µA is awaited. Then, 20 to 50 µL of the analyte is added to the background electrolyte in the cell under continuous stirring. The increase in the concentration of the analyte containing organic peroxide in the electrochemical cell is achieved by successfully adding equal volumes of the analyte to the solution in the electrolysis cell. After each increase in the concentration of the analyte, 5 to 10 s is needed for the signal to reach a steady-state value and for the current strength to be read. The peroxide concentration in the analyte is determined using a previously prepared calibration curve.

### 4.6. Preparation of the Calibration Curve

A calibration curve of BPO at different concentrations was made by preparing a stock solution with concentrations of 0.003 M or 0.3 M in chloroform. To cover the largest possible concentration range, several aliquots of 20 µL and then several rounds of 50 µL of the standard solution were added to the background electrolyte in the cell under continuous stirring at 450–550 rpm. The linear range of the calibration graph was up to 0.2 mM and above, i.e., exceeding a concentration of 48.45 mg/L.

### 4.7. Assessment of the Accuracy of the Method over the Range of Low Peroxide Concentrations

Initially, the catalytic electrode was calibrated with 0.003 M BPO/CHCl_3_ added to fresh oil (anti-cellulite oil—peroxide-free Rivana), according to the following procedure:

1. The preparation of a stock solution with a concentration of 0.03 M BPO in CHCl_3_
2. The weighing of 0.4 g (0.5 mL) of peroxide-free anti-cellulite oil and dissolving it into a 0.03 M BPO/CHCl_3_ solution. This solution is designated as BPO/CHCl_3_/fresh oil.
3. The dilution of 0.03 M BPO/CHCl_3_/fresh oil 10 times with CHCl_3_ to a final concentration of 0.003 M BPO/CHCl_3_/fresh oil.
4. The performance of calibration at a working potential of −0.2 V, with the following portions of 0.003 M BPO/CHCl_3_/fresh oil: 5 × 20 μL. Then, 6 × 50 μL is added.
5. All measurements were performed as described in Section 4.5, strictly following the procedure. The regression equation of the calibration plot is as follows: Y = −2.62 × 10^−9^ + 0.00272x.

Further, standard solutions of benzoyl peroxide (BPO) in chloroform (CHCl_3_) with concentrations of 1, 2, 3, 6, 10 mM were prepared by diluting 0.5 M BPO in CHCl_3_. The standard solutions had a final volume of 10 mL. The signals of all listed standard solutions of BPO in CHCl_3_ were measured. For this purpose, 0.5 mL of each standard solution was taken, and 0.4 g of peroxide-free anti-cellulite oil (Rivana) was dissolved in them according to Table 2.

The aliquot portion taken for analysis from each standard solution was 0.1 mL.

### 4.8. Assessment of the Accuracy of the Electrochemical Method in a Wide Concentration Range

First, a calibration of the catalytic electrode was performed with 0.003 M BPO/CHCl_3_ added to fresh oil (anti-cellulite oil—Rivana, peroxide-free) following the procedure described in Section 4.6.

Then, standard solutions of BPO/CHCl_3_ were prepared with concentrations of 50, 100, 200, 300, 400 mM via the dilution of 0.5 M BPO/CHCl_3_. The standard solutions had a final volume of 2 mL. Their signals were measured, taking 0.5 mL of each standard solution and dissolving ~0.40 g of fresh anti-cellulite oil in them according to Table 3.

The standard solutions from St1 to St5 were pre-diluted and then a 0.1 mL aliquot portion was taken from the already diluted solutions for analysis. The regression equation used for the calibration graph is Y = 2.30 × 10^−8^ + 0.00348x

### 4.9. Peroxide Value (PV) Measurement of the Anti-Cellulite Oil by Iodometric Titration

The PV measurement of oil samples of fresh anti-cellulite oil from the brand Rivana was performed by iodometric titration according to the Bulgarian state standard (BSS EN ISO 3960:2017) [24]. Typically, 0.1000–0.2000 g of vegetable oil is weighed with an accuracy of 0.0002 in a 100 mL Erlenmeyer flask, and 5 mL of chloroform and 2.5 mL of acetic acid are added. The flask is shaken until the oil is completely dissolved, and 1 mL of 50% KI solution (freshly prepared) is added. The sample is left for 5 min in the dark, then 15–20 mL of distilled water and a few drops of 1% starch solution are added until a blue–purple color appears. Then, the sample is titrated with 0.002 N sodium thiosulfate solution slowly and with continuous homogenization by shaking until the color disappears in the aqueous (upper) layer.

For each sample tested, two parallel measurements were made and the arithmetic mean value was taken as the result.

The same procedure was performed for a blank sample, in which only the reagents without vegetable oil are present.

The peroxide value (PV) was calculated according to the following:

$$PV=\frac{\left(V-Vo\right).0.002.1000}{m},meq.O_{2}/kg$$

where:

*V*—volume of 0.002 N Na_2_S_2_O_3_ in cm^3^, used to titrate the vegetable oil sample.

*Vo*—volume of 0.002 N Na_2_S_2_O_3_ in cm^3^, used to titrate the blank sample.

0.002—the concentration in normality of the Na_2_S_2_O_3_ solution

*m*—the mass of vegetable oil

### 4.10. Determination of Peroxide Concentration in a Real Sample—Highly Rancid Anti-Cellulite Oil from the Trademark Rivana

The following procedure was applied:

Four 1 mL samples were taken from the initial solution of expired oil.

An initial standard solution of BPO/CHCl_3_ with a concentration of 10 mM was prepared.

Standard solutions were prepared according to Table 4, and the catalytic electrode was calibrated using the standard addition method.

The signals of all standard solutions were measured, with the aliquot portion used for analysis being 0.1 mL.

### 4.11. Calculus

The intercept in the calibration graph corresponds to the signal of the real sample. Therefore, if we divide this signal by the sensitivity, we will obtain the concentration of peroxides in the sample: 10^−8^/0.008 = 1.25 × 10^−6^ M. Therefore, this signal must be divided by the sensitivity shown in the calibration plot, which gives the concentration of peroxides in the sample: 1 × 10^−8^/0.008 = 1.25 × 10^−6^ M. To account for the dilution of 0.1 mL of sample after its introduction into the cell containing 10 mL of electrolyte, the concentration of peroxides must be multiplied by the dilution factor (DF = 100). Therefore, 1.25 × 10^−6^ M peroxides × 100 = 1.25 × 10^−4^ M peroxides are present in 0.1 mL of sample. Since an injection of the rancid oil (1.300 g) is dissolved with chloroform to a final volume of 15 mL, the concentration of peroxides in this 15 mL of undiluted oil should be found the following way:

1.25 × 10^−4^ M × 0.1 mL of the sample = x × 14.9 mL of sample

x = 1.86 × 10^−2^ M peroxides in 15 mL of sample

The number of moles of peroxides in this 15 mL sample is calculated as follows:

n = M × V = 1 × 86.10^−2^ mol/L × 15 × 10^−3^ L = 2.76 × 10^−4^ mol peroxides.

The number of moles is converted to μmol:

1 mol→106 μmol

2.76 × 10^−4^ mol = 276 μmol

From the data obtained when studying the accuracy of the method for a wider range of PVs, we know that 200 μmol corresponds to PV = 536 meq/kg; then, 276 μmol shall be equal to PV = 739.75 ± 64.65 meq/kg. Therefore, the sample containing a mixture of vegetable oils is very rich in peroxides and is highly peroxidized (rancid).

## 5. Patents

This research resulted in a Bulgarian patent, application No 113803 (BG/P/2023/113803).

## Figures and Tables

**Table 1:** Comparison of the accuracy of the electrochemical method and the classical titrimetric method for the determination of the PV.

Expected PV, meq O_2_/kg (Standard Solution)	Measured PV, meq O_2_/kg, by Titrimetric Method	Deviation of the Titrimetric Value from the Expected, in %	Measured PV, meq O_2_/kg, by Electrochemical Method	Deviation of the Electrochemically Measured PV from the Expected, in %
67	97.0	+44.78	84.2	+25.67
134	165.3	+23.36	153.3	+14.40
268	195.0	−27.24	292.1	+8.99
403	297.4	−26.20	457.5	+13.52
537	417.3	−22.29	565.8	+5.36

**Table 2:** Standards used for calibration of the catalytic peroxide electrode.

Standard Solution	Volume (BPO/CHCl_3_), mL, with the Given Concentration	Mass of the Weighed Oil, g
St1	0.5 mL 1 mM BPO/CHCl_3_	0.4000 ± 0.0075
St2	0.5 mL 2 mM BPO/CHCl_3_	0.4000 ± 0.0075
St3	0.5 mL 3 mM BPO/CHCl_3_	0.4000 ± 0.0075
St4	0.5 mL 6 mM BPO/CHCl_3_	0.4000 ± 0.0075
St5	0.5 mL 10 mM BPO/CHCl_3_	0.4000 ± 0.0075

**Table 3:** Scheme for preparing standard solutions.

Standard Solution	Volume (BPO/Chcl_3_) with Certain Concentration, Ml	DF *	Mass of the Weighed Oil, g
St0	0.5 mL chloroform	1	0.4000 ± 0.0075
St1	0.5 mL 50mM BPO/CHCl_3_	4	0.4000 ± 0.0075
St2	0.5 mL 100 mM BPO/CHCl_3_	10	0.4000 ± 0.0075
St3	0.5 mL 200 mM BPO/CHCl_3_	20	0.4000 ± 0.0075
St4	0.5 mL 300 mM BPO/CHCl_3_	30	0.4000 ± 0.0075
St5	0.5 mL 400 mM BPO/CHCl3	50	0.4000 ± 0.0075

* DF—dilution factor.

**Table 4:** Standard solutions prepared for applying the standard addition method.

Standard Solution	Volume of a Highly Rancid Anti-Cellulite Oil—Rivana, mL	Volume of a Standard 10 mM BPO/CHCl_3_ Solution, mL
St0	0.1	0.000
St1	1.0	0.150
St2	1.0	0.200
St3	1.0	0.250
St4	1.0	0.300

## Data Availability

All data supporting this research are deposited at the Patent Office of the Republic of Bulgaria (application No BG/P/2023/113803).
